# Anti-allergic activity of glycyrrhizic acid on IgE-mediated allergic reaction by regulation of allergy-related immune cells

**DOI:** 10.1038/s41598-017-07833-1

**Published:** 2017-08-03

**Authors:** Shiwen Han, Lu Sun, Feng He, Huilian Che

**Affiliations:** 1 Beijing Advanced Innovation Center for Food Nutrition and Human Health, College of Food Science and Nutritional Engineering, China Agricultural University, Beijing, 100083 P. R. China; 2 College of Food Science and Nutritional Engineering, China Agricultural University, Beijing, 100083 P. R. China

## Abstract

Glycyrrhizic acid (GA), the major bioactive triterpene glycoside of glycyrrhiza, has been shown to possess a wide range of pharmacological properties, including anti-inflammatory and anti-viral properties. However, few studies have examined the anti-allergic activity and exact mechanism of action of GA. In the present work, the anti-allergic activity and possible mechanisms of action of GA on an immunoglobulin (Ig) E-mediated allergic reaction has been studied using three models of allergic reaction *in vivo* and *in vitro*. Active systemic allergic reaction in Balb/c mice showed that GA can suppress the increased level of IL-4 to restore the immune balance of T_H_1/T_H_2 cells in a dose-dependent manner. Additionally, GA attenuated significantly the B cells producing allergen-specific IgE and IgG_1_ partly because of the low levels of T_H_2 cytokines. Both passive cutaneous anaphylaxis *in vivo* and an RBL-2H3 cell-based immunological assay *in vitro* indicated that GA acted as a “mast cell stabilizer”, as it inhibited mast cell degranulation and decreased vascular permeability by inhibiting the expression of Orai1, STIM1 and TRPC1, which blocked extracellular Ca^2+^ influxes. The current study suggests that GA may serve as an effective anti-allergic agent derived from food for the prevention and treatment of IgE-mediated allergic reaction.

## Introduction

An allergic condition describes a hypersensitivity disorder in which the immune system reacts to substances in the environment that are normally considered harmless^1^. This rapid-onset, potentially life-threatening disease is common worldwide with a high prevalence reported in all age groups^2^. Anaphylaxis can be most commonly triggered by exposure to allergens, such as insect venoms, foods and medications, through skin contact, injection, ingestion or inhalation^3^. One of the most important health problems is food allergy. It was reported that food allergy, associated with nausea, vomiting, diarrhoea, peptic ulcers, asthma, allergic dermatitis, allergic rhinitis, allergic shock and even death, commonly triggered by immunoglobulin (Ig) E^4^ has seriously affected nearly 5% of adults and 8% of children in developed countries annually. Its incidence manifests a rising tendency with each passing year^5^. Thus far, there are no therapies available to cure allergic diseases completely. Some medicines, such as anti-histamine drugs (diphenhydramine, chlorpheniramine maleate, terfenadine, etc.), mast cell stabilizers (disodium cromoglycate, sodium hydroxypropylcromate, ketotifen, etc.) and immune suppressors (adrenal cortical hormones, dexamethasone, hydrocortisone, etc.), can only be used to help relieve allergic symptoms and alleviate the suffering of anaphylaxis. However, these drugs not only have side effects, but do not prevent symptom relapse. Surely, anti-allergic ingredients derived from food without side effects and relapse would be a suitable alternative anti-allergic strategy.

Many studies have found that biologically active ingredients of natural foods with antioxidant or anti-inflammatory properties, such as flavonoids and polyphenols, contribute to anti-allergic activity^6–11^. Glycyrrhiza is a plant of ancient origin, and its main component, glycyrrhizic acid (GA)^12^, has been widely used in foods and traditional herbal medicines^13^. Clinical and experimental studies suggest that GA possesses several useful pharmacological properties, including anti-inflammatory^14^ and immunomodulatory^15^ properties. In a Balb/c mouse asthma model, GA (2.5–20 mg/kg∙bw) can prevent the reduction of interferon (IFN)-γ and total IgG_2a_ levels and also decrease interleukin (IL)-4, IL-5, eosinophilia and OVA-specific IgE^16^. In addition, GA (10 mg/kg∙bw) can attenuate the development of carrageenan-induced acute inflammation by preventing the activation of NF-κB and STAT-3^17^. Based upon these observations, we hypothesized that GA might be a contributing factor in the medicinal or nutritional uses of glycyrrhiza for relieving allergic reaction. However, few reports are available on the anti-allergic activity of GA. The present study was designed to investigate the anti-allergic effect of GA and to explore its possible underlying mechanism using active systemic allergic reaction and passive cutaneous anaphylaxis *in vivo* and an RBL-2H3 cell-based immunological assay *in vitro*.

## Results

### GA reduces OVA–induced systemic allergic reaction in Balb/c mice through the regulation of T-helper (Th) cell differentiation

**Figure 1:**
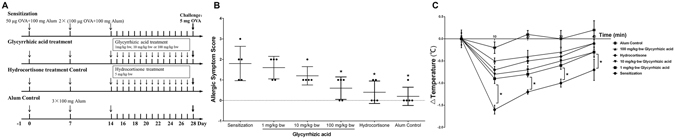
GA attenuated clinical allergic symptoms and the variation of rectal temperature in Balb/c mice. ^*^ *P* < 0.05 as compared to the sensitization group (n = 5). (**A**) Schematic drawing representing the Balb/c mice active system food anaphylaxis protocols and doses used in this work. (**B**) Allergic symptom score and (**C**) The variation of rectal temperature (°C).

To assess the anti-allergic effect of GA on the IgE-mediated allergic reaction, we examined an active systemic allergic reaction in Balb/c mice. OVA-induced food allergy symptoms were evaluated and scored for allergic symptoms and rectal temperature after a challenge for 40 min. Several allergic symptoms of OVA-induced food allergy were observed in the sensitization group, including strongly reduced activity, scratching, bristled fur and sometimes laboured respiration (1.80 ± 0.84 points). In contrast, the 100 mg/kg∙bw GA-treated group showed significant suppression of the allergic symptoms (0.60 ± 0.55 points, Fig. 1B). In addition, the rectal temperature in the sensitization group decreased by −1.60 ± 0.1 °C compared to the Alum control group, whereas in the 1 mg/kg∙bw GA-treated group, the rectal temperature was only reduced by −0.90 ± 0.1 °C (Fig. 1C). The suppressive effect of 100 mg/kg∙bw of GA was similar to that of hydrocortisone, a common drug for the treatment of anaphylaxis, which was used as a positive control.

**Figure 2:**
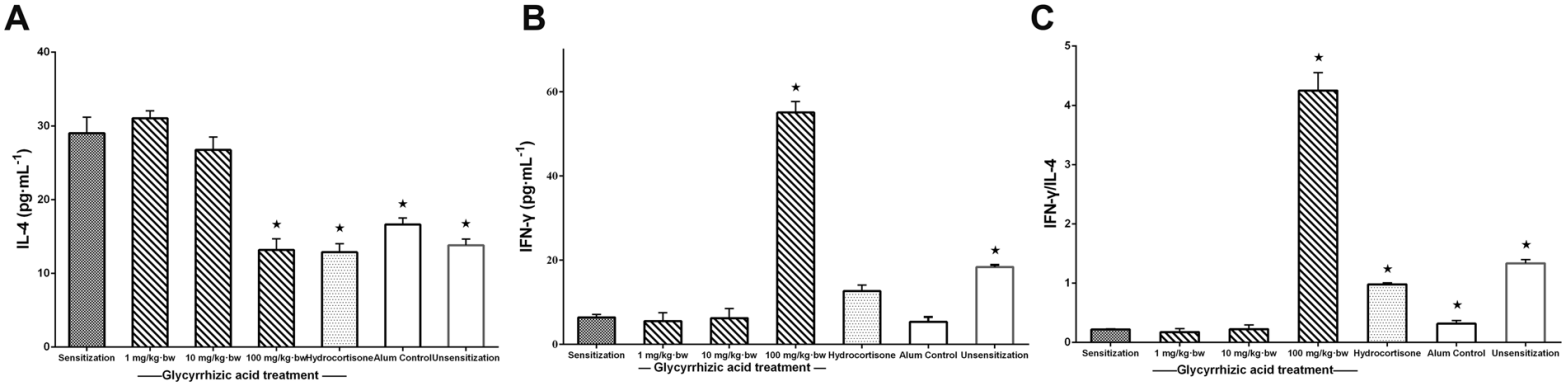
GA worked on T_H_ cells to modulate the T_H_1/T_H_2 subsets balance. ^*^ *P* < 0.05 as compared to the sensitization group (n = 5). (**A**) Concentration of IL-4 and (**B**) IFN-γ in spleen cells. (**C**) The ratio of IFN-γ and IL-4.

We then investigated the cytokine patterns in mouse spleen cells. T_H_2-related cytokine (IL-4) was increased by the allergy induction and inhibited by GA, especially at the dose of 100 mg/kg∙bw of GA (Fig. 2A). T_H_1-related cytokine (IFN-γ) decreased in the sensitization group, the GA group (1 or 10 mg/kg∙bw) and the hydrocortisone group compared to the non-sensitized group. However, it was significantly increased by 100 mg/kg∙bw of GA compared to the levels observed in the sensitization group (*P* < 0.05, Fig. 2B). Alum, which can activate T_H_2-type immune cells^18^, also decreased the IFN-γ level (Fig. 2B). The result for IFN-γ/IL-4 was similar to that of IFN-γ (Fig. 2C), and 100 mg/kg∙bw of GA ultimately results in a T_H_1-type immune response. These results demonstrated that an oral dose of 1–100 mg/kg∙bw of GA may affect T_H_ cells by modulating the T_H_1/T_H_2 immune balance, thus attenuating the allergic reaction. A high concentration of GA can also affect the immune balance.

### GA inhibits OVA-specific IgE and IgG_1_ production by affecting OVA-specific antibody-producing B cells

**Figure 3:**
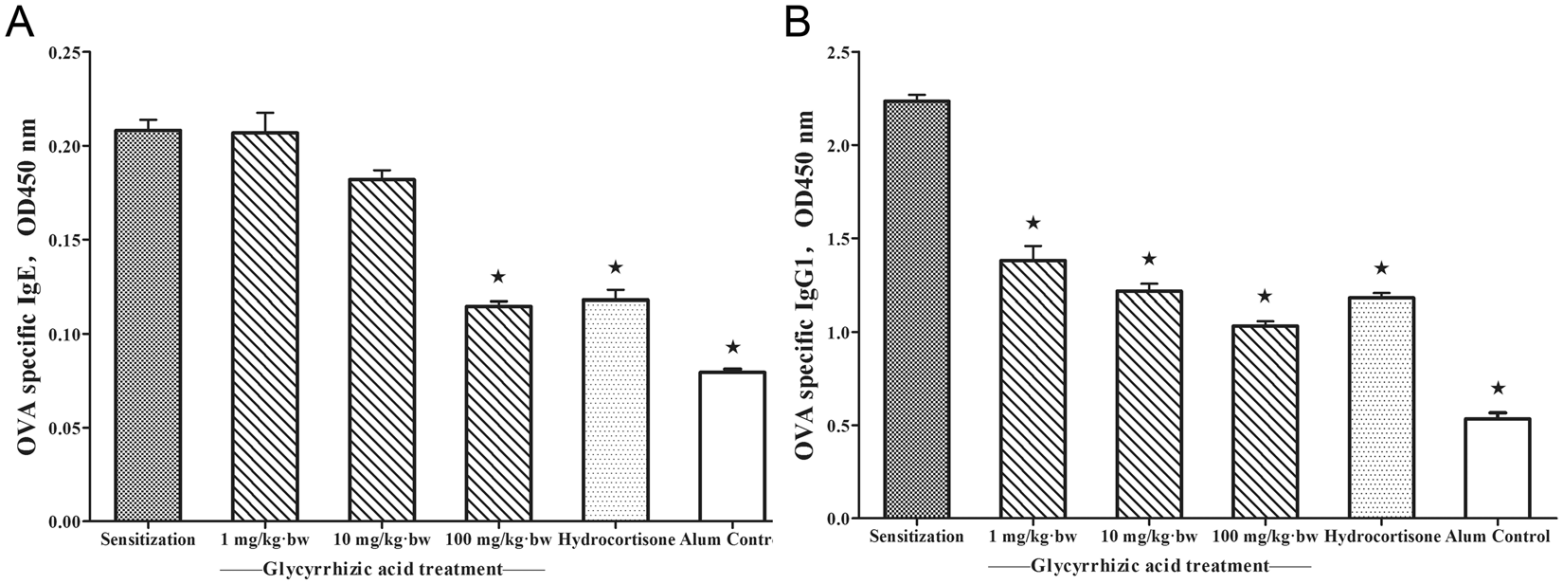
GA inhibited the production of OVA-specific IgE and IgG_1_ from B cells. ^*^ *P* < 0.05 as compared to the sensitization group (n = 5). The level of OVA-specific (**A**) IgE and (**B**) IgG_1_ in serum.

We next investigated the effect of GA on the production of IgE and IgG_1_, the T_H_2-type antibodies, against the OVA. The production of OVA-specific IgE and IgG_1_ was significantly increased in the sensitization group compared to the Alum control group (*P* < 0.05) and inhibited by GA (Fig. 3A and B). Only 100 mg/kg∙bw can significant decrease the OVA-specific IgE production (*P* < 0.05, Fig. 3A). The significant inhibitory activity of GA against the OVA-specific IgE and IgG_1_ production was similar to that of hydrocortisone. These results demonstrated that GA also influenced OVA-specific antibody-producing B cells.

### GA can also act as a “mast cell stabilizer” to relieve allergic symptoms by suppressing mast cell-mediator release

**Figure 4:**
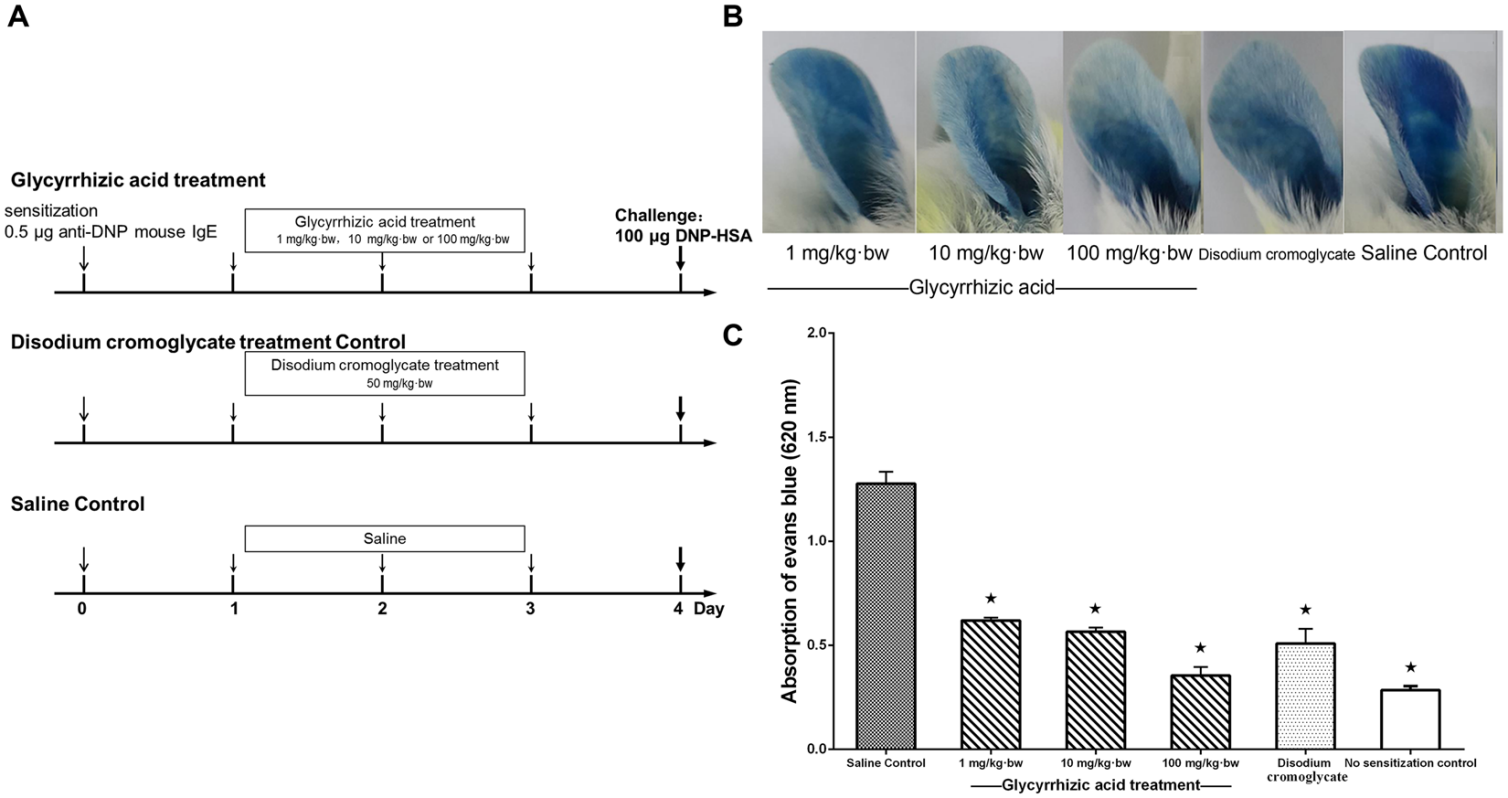
GA attenuated the vascular permeability by stabilizing mast cells. ^*^ *P* < 0.05 as compared to the saline control group (n = 5). (**A**) Schematic drawing representing the Balb/c mice passive cutaneous anaphylaxis protocols and doses used in this work. (**B**) Qualitative and (**C**) quantitative detection of the vascular permeability after Evan’s Blue Dye/DNP-HSA administration.

Mast cells are responsible for IgE-induced anaphylaxis^19^ through the secretion of various inflammatory cytokines and mediators that can strengthen allergic symptoms. We then tested whether GA also regulates mast cell activation using passive cutaneous anaphylaxis (PCA) and an RBL-2H3 cell-based immunologic assay. GA significantly attenuated the mast cell-dependent PCA reaction in a dose-dependent manner, exhibiting 49.1%, 47.1% and 26.9% suppression at 1, 10 and 100 mg/kg∙bw of GA based upon Evans blue extravasation, respectively (*P* < 0.05, Fig. 4B and C). Both the quantitative and qualitative PCA results indicated that GA can inhibit the decreased vascular permeability to reduce the albumin leakage; this effect is similar to the sodium cromoglycate.

**Figure 5:**
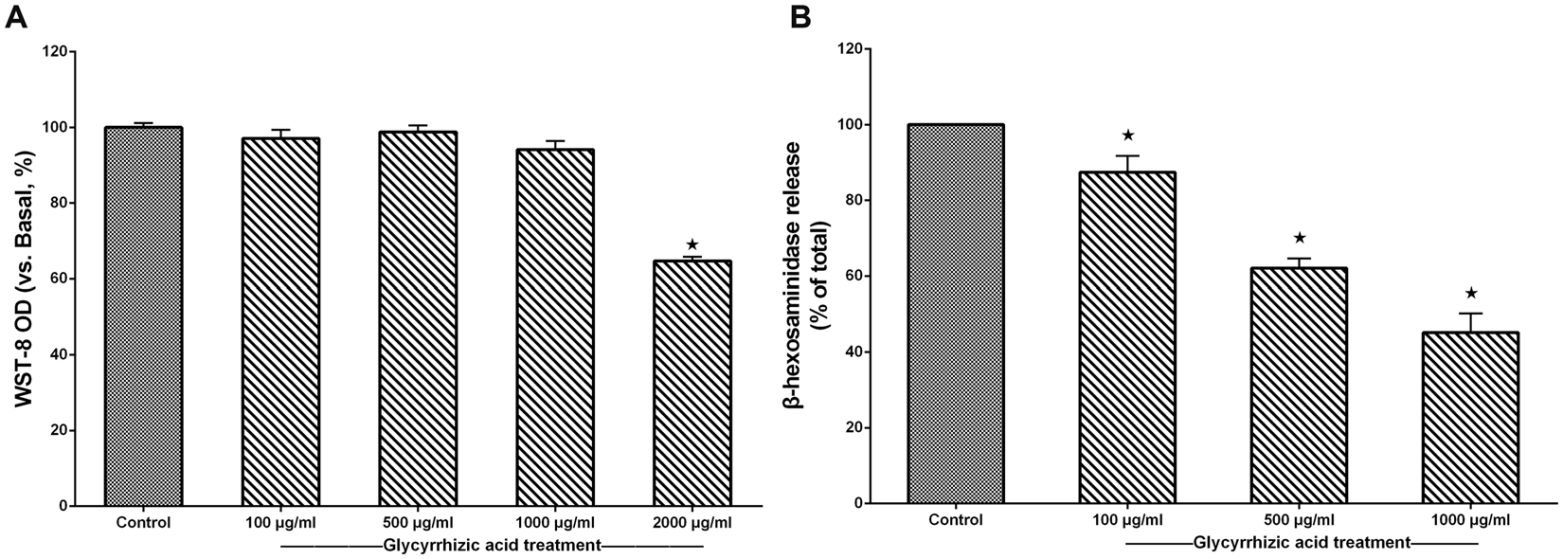
GA inhibited the degranulation of RBL-2H3 cells. ^*^ *P* < 0.05 as compared to the control group (n = 3). (**A**) WST-8 cell viability (%) assay for GA at various concentration (100, 500, 1000, 2000 μg/mL). (**B**) β-hexosaminidase release (%) of RBL-2H3 cells after GA treatment.

Similar results with GA treatment were also obtained using the RBL-2H3 cell assay. We first examined the cytotoxic effect of GA on RBL-2H3 cells using the WST-8 assay and found that GA did not affect cell viability at 100~1000 μg/mL (Fig. 5A). Therefore, concentrations of GA < 1000 μg/mL were used for subsequent experiments. To investigate the effect of GA on degranulation, we measured the release of β-hexosaminidase in the presence or absence of GA. GA strongly suppressed β-hexosaminidase release from 87.46% ± 7.52% to 45.23% ± 8.64% as the dose of GA increased from 100 to 1000 μg/mL (*P* < 0.05, Fig. 5B).

### GA stabilizes mast cells by reducing the expression of the calcium channel proteins

**Figure 6:**
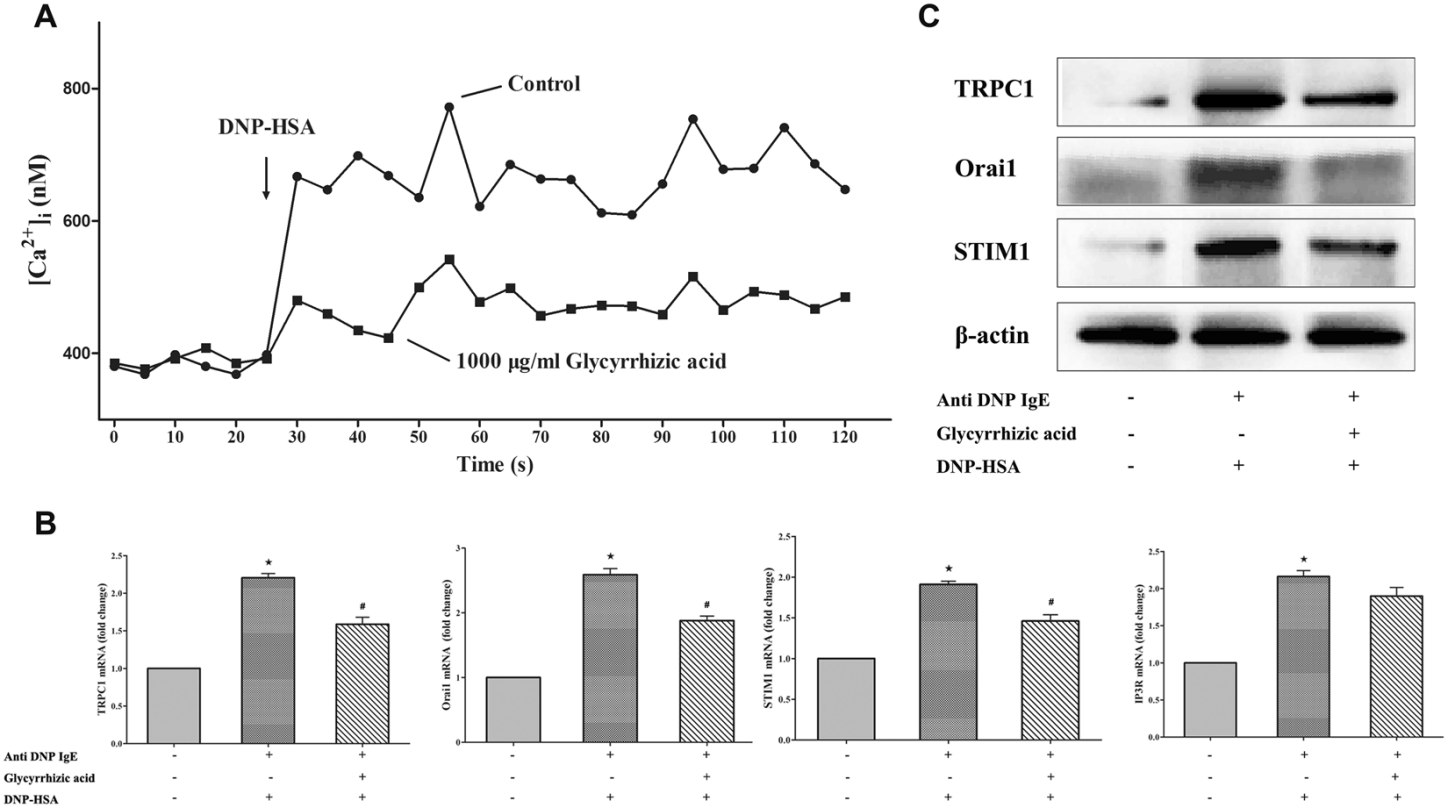
GA blocked the exCa^2+^ influx to stabilize RBL-2H3 through reducing calcium channel proteins expression. ^*^ *P* < 0.05, significantly different from control and ^#^ *P* < 0.05, significantly different from sensitization control without GA treatment (n = 3). (**A**) Effect of GA on [Ca^2+^]_i_. (**B**) The mRNA relative expression of Orai1, STIM1, TRPC1 and IP3R. (**C**) The protein expression of calcium channel proteins.

As reported, the degranulation of RBL-2H3 cells depends on Ca^2+^ release from the endoplasmic reticulum (ER) and calcium release-activated calcium (CRAC)-mediated Ca^2+^ influxes^20^. We further investigated the effect of GA on Ca^2+^ influx. Fluo-3AM, a fluorescent Ca^2+^ indicator, was used to determine the intracellular Ca^2+^ concentration. A significant increase of [Ca^2+^]_i_ (nM, intracellular Ca^2+^ concentration) was observed after DNP-HSA challenge at 30 s, and 1000 μg/mL of GA completely inhibited IgE/Ag-stimulated Ca^2+^ influx (Fig. 6A).

We further investigated the expression of Ca^2+^ influx-related proteins (calcium release-activated calcium channel protein 1 (Orai1), stromal interaction molecule 1 (STIM1), transient receptor potential channel 1 (TRPC1) and inositol 1, 4,5-trisphosphate receptor (IP3R)). Based upon RT-PCR and Western blotting results, the expression levels of Orai1, STIM1 and TRPC1 were significantly decreased by GA (*P* < 0.05, Fig. 6B and C, ﻿Fig. S1). IP3R, a receptor expressed on the ER membrane, was unaffected by GA at the mRNA level (*P* > 0.05, Fig. 6B). We then confirmed that GA had no effect on the depletion of ER calcium store, but can stabilize mast cells by inhibiting the Ca^2+^ influx due to the lower expression of calcium channel proteins (Orai1, STIM1 and TRPC1).

## Discussion

**Figure 7:**
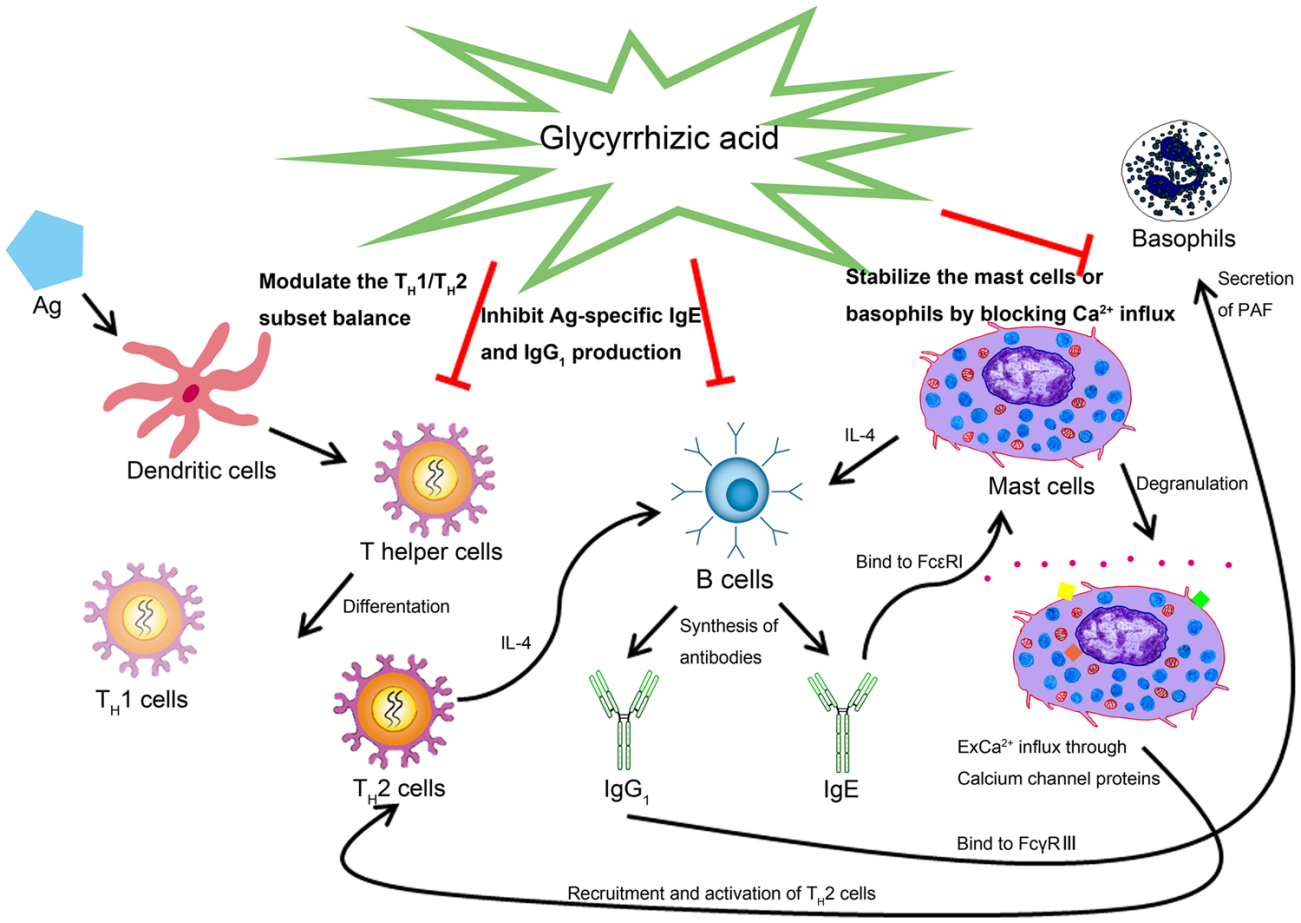
The mechanism of anti-allergic effect of GA on the IgE-mediated allergic reaction.

Natural triterpenoid compounds, such as glycyrrhizic acid (GA), ursolic acid, oleanolic acid and nomilin, exert similar effects on the immune system of Balb/c mice^12^, which may be related to the similarities in their chemical structures. In addition to the anti-inflammatory, anti-viral, antineoplastic and immune regulatory pharmacological effects, GA was found to possess anti-allergic activity in our study. The three main mechanisms of anti-allergic effect of GA are summarized in Fig. 7: GA (1) regulates the T_H_ cell differentiation, which decreased the elevated level of secretion of T_H_2-related cytokine (IL-4) to restore T_H_1/T_H_2 immune balance; (2) affects the OVA-specific antibody producing B cells; and (3) acts as a “mast cell stabilizer” to reduce mediator release through the inhibitory effect of Ca^2+^ influx due to the lower expression of calcium channel proteins.

Many reports have identified that GA can affect the secretion of cytokines to modulate the immune microenvironment. In a cell-mediated immune response, GA (313 pg/ml) triggered a reduction of the highly elevated level of IL-6 compared with the control animals. In contrast, the level of IL-2 was enhanced with 37.9 pg/ml of GA in metastatic tumour-bearing C57BL/6 mice^21^. Moreover, GA still enhanced IFN-γ levels and reduced IL-4 levels in an allergic rhinitis mouse model^22^, which points to many biological roles including suppressing the stimulation of activated B-cell and T-cell proliferation and the differentiation of CD4^+^ T cells into T_H_2 cells^23^. In a murine model of asthma, GA exerted a therapeutic effect on OVA-induced experimental asthma partly by regulating the T_H_1/T_H_2 balance through suppressing OX40-OX40 L signalling and p38 MAPK activity^24^. The results of above reports are consistent with our study, which identified the modulatory effect of GA on T_H_ cells. Furthermore, we found that a high concentration of GA (100 mg/kg∙bw) triggers a T_H_1-type immune response.

As previously reported, except for its regulatory effect on the T_H_1/T_H_2 immune balance, GA can reduce total IgE and OVA-specific IgE levels in serum^24^. OVA-specific IgE was decreased significantly in a dose-dependent manner after GA treatment in an allergic rhinitis mouse model, which may be induced by inhibiting T_H_2 cell differentiation and maturation, and IL-4 production subsequently prevented allergic rhinitis development^22^. That GA can suppress the production of T_H_2 antibodies (IgE and IgG_1_) from OVA-specific antibody producing B cells is probably because of the effect of GA on the T_H_ cell differentiation. GA produced a more significant suppressive effect on IgG_1_, which may subsequently inhibit the IgG_1_-mediated basophil activation^25^.

Previous studies have found that GA can inhibit histamine synthesis and release in mast cells co-cultured with Swiss 3T3 fibroblasts^26^. In our study, passive cutaneous anaphylaxis, which mainly depends on mast cells *in vivo*, showed that GA significantly reduced vascular permeability in a way similar to sodium cromoglycate. Similarly, GA can inhibit the release of β-hexosaminidase, a biomarker of degranulation, in RBL-2H3 cells. Our findings confirmed that GA acts as a “mast cell stabilizer” by inhibiting mast cell-mediator release.

Ca^2+^ is an essential cofactor for the degranulation of RBL-2H3 cells^27^, which can regulate the granule-plasma membrane fusion and the release of mediators^28^. Based upon [Ca^2+^]_i_ measurement, the increased level of intracellular Ca^2+^ concentration was significantly reduced after GA treatment, which suggested that GA also plays a stabilizing role on mast cells by inhibiting the extracellular Ca^2+^ influx process. No difference in the mRNA expression of IP3R in the presence or absence of GA was found, which indicated that GA has no effect on the depletion of ER Ca^2+^ store. The decreased expression of Orai1, STIM1 and TRPC1 both at the mRNA and protein levels indicate that GA might suppress the Ca^2+^-dependent degranulation due to the lower expression of these calcium channel proteins.

Based on the combined *in vitro* and *in vivo* analysis of GA treatment, we can conclude that GA exerts an anti-allergic effect by influencing T_H_ helper cells, OVA-specific antibody-producing B cells and mast cells (or basophils) (Fig. 7). After the allergen is captured by dendritic cells through the disrupted epithelium, allergen-activated dendritic cells mature and migrate to regional lymph nodes where they present processed allergen epitopes to cognate T cells. Such T cells differentiate and become activated T_H_2 cells, but GA can suppress this process to restore the T_H_1/T_H_2 immune balance. IL-4, which may be derived from T_H_2 cells, mast cells, and basophils, also activates immunoglobulin heavy chain gene CSR for allergen-specific IgE production^29^. However, GA inhibits the synthesis and production of OVA-specific IgE and IgG_1_ from the antibody producing B cells. Allergen-specific IgE can bind to FcεRI to stimulate mast cell degranulation^30^ and to FcγRIII to activate PAF release from basophils^19^; these processes recruit and activate T_H_2 cells^31^ to begin a positive feedback loop. However, GA, as a “stabilizer”, reduces the release of allergic mediators by blocking extracellular Ca^2+^ influxes due to the lower expression of calcium channel proteins (Orai1, STIM1 and TRPC1). In conclusion, as confirmed by active systemic allergic reaction, passive cutaneous anaphylaxis and RBL-2H3 cell-based immunology assay, GA exerts anti-allergic activity and can be used as a potential anti-allergic nutrient in the future.

## Materials and Methods

### Drugs and chemicals

The chemicals were obtained from the following suppliers: Glycyrrhizic acid (GA), Purity >98% (G111375) and hydrocortisone (H110523) (Aladdin biochemical technology Co. Ltd., China); Ovalbumin (A5503), sodium cromoglycate (C0399), monoclonal anti-Dinitrophenyl antibody produced in mouse (anti-DNP IgE, D8406), dinitrophenol-human serum albumin (DNP-HSA), HEPES (H3375), water-soluble tetrazolium-8 (WST-8, 96992), 4-nitrophenyl N-acetyl-b-D-glucosaminide (N9376), Evans blue (E2129) and Fluo-3AM (39294) (Sigma–Aldrich Corp., USA); Imject™ Alum Adjuvant (77161, Thermo Fisher Scientific Inc., NY). TransScript One-Step gDNA Removal and cDNA Synthesis SuperMix (AT311) and TransStart Top Green qPCR SuperMix (AQ131) (TransGen Biotech, China). HRP-tagged goat anti mouse IgG_1_(ab97240), HRP-tagged goat anti mouse IgE (ab11580), Anti-TRPC-1 antibody (ab192031), Anti-Orai1 antibody (ab83751) and Anti-Stromal interaction molecule 1 antibody (ab108994) (Abcam, UK). Anti-rabbit IgG HRP-linked Antibody (#7074 S, Cell Signaling Technology Inc., USA). All other chemicals and solvents used in this study were of analytical grade.

### Animals and management

Balb/c mice in our research were obtained from Vital River Laboratories, Inc. (Beijing, China). The study was conducted in the specific pathogen free (SPF) animal laboratory of College of Food Science and Nutritional Engineering, China Agricultural University (Beijing, China). Animal rooms were maintained with temperature of 22 ± 1 °C, humidity of 55 ± 5%, a 12 h light/dark cycles and air exchanges at 15 times/h. Feed and water were supplied *ad libitum*. The commercial SPF rodent maintenance feed produced by Ke Ao Xie Li feed Co. Ltd. (Beijing, China) met the Chinese Standard GB14924.3–2010. Animal experiments in our research were carried out in accordance with the Guide for the Animal Experimental Welfare and Ethical in the Food Science and Nutritional Engineering College of China Agricultural University and were approved by the Animal Experimental Welfare and Ethical Inspection Committee in China Agricultural University. All efforts were made during the animal experiments to minimize suffering.

### Establishment of active systemic allergic reaction in Balb/c mice

Thirty female Balb/c mice (4-weeks old, weighing 18–22 g) were divided into six groups (n = 5) with an initial body weight difference of ±20% after 3 day acclimation. The mice in the Alum immune adjuvant control group, were injected intraperitoneally with 200 μL of Imject^TM^ Alum Adjuvant (100 mg in 0.9% NaCl) on day 0, 7 and 14. Mice in the sensitization group, GA treatment group and hydrocortisone treatment control group were sensitized by intraperitoneal injection of 200 μL of OVA solution (50 μg of OVA and 100 mg of Imject^TM^ Alum Adjuvant in 0.9% NaCl) on day 0. The second and third sensitizing doses of OVA were increased to 100 μg on day 7 and 14. Finally, the sensitized mice were challenged intragastrically with a high dose of OVA (5 mg of OVA in 0.9% NaCl) on day 28. Before the challenge, GA was administered orally at a concentration of 1 mg/kg∙bw, 10 mg/kg∙bw or 100 mg/kg∙bw daily between day 16 to day 27. A dose of 5 mg/kg∙bw of hydrocortisone, a common drug for treating allergy diseases, was used as a treatment control. The experimental treatment design is shown in Fig. 1A.

### Clinical allergic symptoms score system and rectal temperature

We first determined the anti-allergic effect of GA based upon the clinical allergic symptom score system and rectal temperature. The clinical allergic symptoms were scored 30 min post-challenge as previously described^32^: 0, no signs; 1, mice are scratching between 4 and 10 times over 15 min; 2, mice are scratching more than 10 times over 15 min, or display reduced activity or bristled fur; 3, mice have a strongly reduced activity, watery diarrhoea, difficulty walking normally, bristled fur and sometimes laboured respiration; 4, similar to degree 3 but stronger with cyanosis around the mouth and tail; and 5, death. Rectal temperature was measured before and 40 min after the challenge using a WI88375 probe (Beijing Science and Technology, Beijing, China).

### ELISA assays for serum OVA-specific antibodies and spleen cell cytokines

To measure the serum OVA-specific IgE and IgG_1_ levels by ELISA as previously described^33^, we collected serum samples from the orbital sinus after challenge with 5 mg OVA on day 28. Meanwhile, spleens were isolated from mice under sterile conditions after sacrifice, and spleen cells were seeded at 2 × 10^5^ cells/well on a 96-well cell culture plate and incubated in RPMI1640 medium containing 200 μg/mL of OVA for 72 h at 37 °C in a 5% CO_2_ incubator. Cytokines were quantified using a commercial mouse ELISA kit (eBioscience, Inc., San Diego, CA).

### Establishment of passive cutaneous anaphylaxis in Balb/c mice

Twenty-five female Balb/c mice (4-weeks old, weighing 18–22 g) were divided into five groups (n = 5). All tested mice received an intradermal injection of 0.5 μg of anti-DNP IgE in 30 μL of saline in the right ear and 30 μL of saline only in the left ear. On day 2, 3 and 4, Balb/c mice were administered orally 1 mg/kg∙bw, 10 mg/kg∙bw or 100 mg/kg∙bw of GA. At the same time, 50 mg/kg∙bw of disodium cromoglycate was administered orally as the treatment control. On day 5, each mouse was injected intraperitoneally with 200 μL of DNP-HSA and Evans blue solution (100 μg DNP-HSA and 2% Evans blue in 0.9% NaCl). The experimental treatment design is summarized in Fig. 4A.

### Evans blue extravasation assay in Balb/c mice ears

After challenge, Evans blue extravasation in the right ears was recorded by a Canon EOS camera to qualitatively analysis the vascular permeability. After mice were sacrificed at 50 min, ears were collected and incubated with formamide at 64 °C for 12 hours. The concentrations were determined at 620 nm using the Thermo Scientific Varioskan Flash (Thermo, USA) to quantitatively evaluate the vascular permeability.

### RBL-2H3 cells culture

The rat basophilic leukemia cell line (RBL-2H3) obtained from National platform of experimental cell resources (Beijing, China) was cultured in MEM medium supplemented with 15% fetal bovine serum (FBS) and 1 × 10^5^ U/L penicillin/streptomycin at 37 °C in a humidified 5% CO_2_ incubator.

### Water-soluble tetrazolium-8 (WST-8) cell viability assay

RBL-2H3 cells were preincubated with or without GA at a final concentration of 100 μg/mL, 500 μg/mL, 1000 μg/mL or 2000 μg/mL for 24 h. After washing the cells 3 times with PBS, 10 μL of WST-8 was added for incubating another 1 h at 37  °C. Finally, supernatants were transferred into another 96-well plate for measurement at 450 nm with the Thermo Scientific Varioskan Flash (Thermo, USA).

### RBL-2H3 cell-based immunological assay

Firstly, the cells were preincubated with 1 μg/mL anti-DNP IgE for 2 h. After washing again with PBS, GA at a final concentration of 0, 100, 500 or 1000 μg/mL was added to incubate for 20 min. Finally, the cells were stimulated with 50 μL DNP-HSA (100 ng/ml) for 45 min at 37 °C.

### β-hexosaminidase release assay

After stimulation with DNP-HSA, 30 μL of supernatant were transferred to a 96-well plate and incubated with 50 μL of p-Nitrophenyl-N-Acetyl-β-D-Glucosaminide (1.3 mg/mL in 0.1 M citric acid buffer, pH 4.5) for 1 h at 37 °C. The reaction was stopped by adding 200 μL stop solution (0.1 M Na_2_CO_3_/NaHCO_3_, pH 10.0). The absorbance of each well was measured at 405 nm using a Thermo Scientific Varioskan Flash microtiter plate reader (Thermo, USA). The total release of β-hexosaminidase was determined in RBL-2H3 cells without GA and the spontaneous release of RBL-2H3 cells was evaluated by adding 50 μL of Tyrode’s buffer instead of DNP-HSA to each well. The release of β-hexosaminidase was calculated as follows (Equation 1).

1 $$\begin{array}{c}{\rm{\beta }} \mbox{-} \mathrm{hexosaminidase}\,{\rm{release}}\,(100 \% )\\ =\,\frac{absorbance\,of\,test\,samples-absorbance\,of\,Tyrode\mbox{'}s\,solution}{absorbance\,of\,total\,release-absorbance\,of\,Tyrode\mbox{'}s\,solution}\times 100 \% \end{array}$$

### Measurement of intracellular Ca^2+^ concentration

Cells were seeded into a 96-well black opaque cell culture plate. After sensitization with mouse monoclonal anti-DNP IgE and treatment with or without GA, cells were incubated with 5 μM of Fluo-3AM for 30 min at 37 °C in the dark and free Fluo-3AM was removed by washing. Following a 30 s baseline recording, cells were exposed to 100 ng/mL of DNP-HSA for another 300 s. FI (fluorescence intensity) was recorded using the Thermo Scientific Varioskan Flash microtiter plate reader with excitation at 488 nm and emission at 525 nm. [Ca^2+^]_i_ was calculated as follows (Equation 2)^34^:

2 $${[{{\rm{Ca}}}^{2+}]}_{{\rm{i}}}({\rm{nm}})={{\rm{K}}}_{{\rm{d}}}[({{\rm{F}}-{\rm{F}}}_{{\rm{\min }}})/({{\rm{F}}}_{{\rm{\max }}}-{\rm{F}})]$$

where F_min_ is the background fluorescence with 5 mM EGTA and F_max_ is the maximum fluorescence with 0.1% Triton X-100 instead of DNP-HSA. K_d_, the effective dissociation constant, of Fluo-3 and Ca^2+^ is 400 nM.

### RT-PCR analysis

Total RNA was prepared using Trizol reagent and cDNA was transcribed using the TransScript One-Step gDNA Removal and cDNA Synthesis SuperMix. RT-PCR was performed using the TransStart Top Green qPCR SuperMix for STIM1, Orai1, TRPC1, IP3R and β-actin. PCR for RBL-2H3 was performed with primers as follows:

5′-ATGCCACGTCTTCCAATGGT-3′ and 5′-TCAGCCATAGCCTTCTTGCC-3′ for STIM1, 5′-GCCATAAGACGGACCGACAG-3′ and 5′-ACTTAGGCATAGTGGGTGCC-3′ for Orai1, 5′-AGCTGCTTATCTTCATGTGCG-3′ and 5′-AGCACGAGGCCAGTTTTGTA-3′ for TRPC1, 5′-AGCATCTCCTTCAACCTGGC-3′ and 5′-CACAGTTGCCCACAAAGCTC-3′ for IP3R and 5′-GCAGGAGTACGATGAGTCCG-3′ and 5′-ACGCAGCTCAGTAACAGTCC-3′ for β-actin. The 2^−ΔΔCt^ method was used to calculate the relative mRNA levels, and β-actin was used as the internal control.

### Western Blotting

The cell samples were homogenized in a lysis buffer with protease inhibitors. The protein concentration of the supernatant was determined using a BCA Protein Assay Kit. Protein samples (40 μg) from different experimental groups were separated by SDS-PAGE (10%), transferred to nitrocellulose membranes, blocked in TBST solution containing 5% BSA for 1 h at room temperature, and incubated overnight at 4 °C with antibodies against STIM1, Orai1, TRPC1 or β-actin. After washing 6 times with TBST, the membranes were next incubated with HRP-conjugated secondary antibody for 1 h at room temperature. The signal was visualized by enhanced chemiluminescence and exposure to an X-ray film (Sage creation Mnin Chemi II, China).

### Statistical analyze

Statistical significance was determined by one-way analysis of variance (ANOVA) using GraphPad Prism 5.01 (GraphPad Software, Inc., USA). All data are presented as the mean values ± standard deviation (SD) with three times biological replicates and statistical significance was set at P-value < 0.05.

## Electronic supplementary material

Supplementary Figure 1
