# Supplementary material for: Anti-allergic activity of glycyrrhizic acid on IgE-mediated allergic reaction by regulation of allergy-related immune cells
Source: Sci Rep. 2017 Aug 3;7:7222. doi: 10.1038/s41598-017-07833-1 (PMC5543155; doi:10.1038/s41598-017-07833-1)
Supplement: Supplementary file 1 — Supplementary Figure 1 [file 41598_2017_7833_MOESM1_ESM.pdf]

Anti-allergic activity of glycyrrhizic acid on IgE-mediated anaphylaxis via regulation of the allergy-related immune cells

Shiwen Han[a], Lu Sun[b], Feng He[b], Huilian Che[a*]

[a] *Beijing Advanced Innovation Center for Food Nutrition and Human Health, College of Food Science and Nutritional Engineering, China Agricultural University, Beijing 100083, P.R. China;*

[b] *College of Food Science and Nutritional Engineering, China Agricultural University, Beijing 100083, P.R. China.*

[*]Corresponding author's email address: chehuilian@cau.edu.cn.

Funding sources: This work was supported by National Natural Science Foundation of China under grant No. 81573158.

Supplementary Information

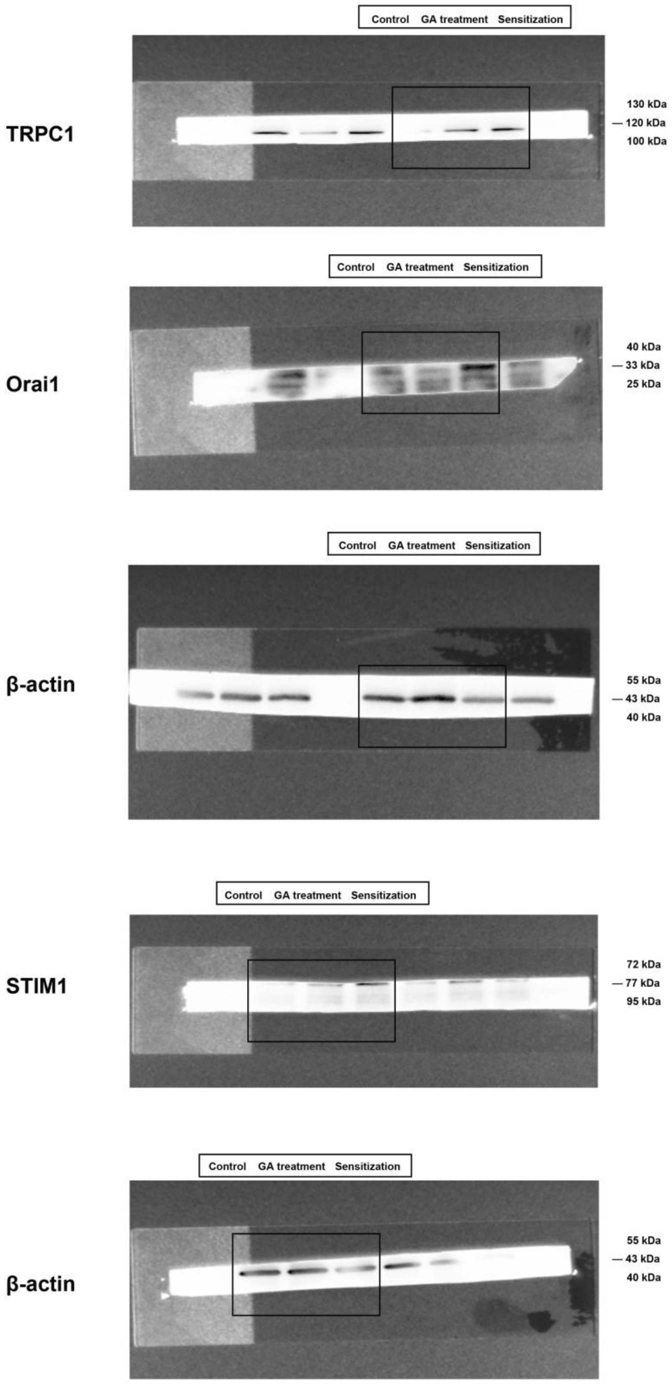

Supplementary Fig.1 Full-length blots of Fig.6C.

All of the blots were the first exposure in this Fig. and the NC membranes were tailored according to the suitable protein molecule weight prior to incubating with primary antibody.
